# On the Relation of Medicine to Surgery; and on the Division of Medical Practitioners into Two Grades, &c.

**Published:** 1842-10

**Authors:** 


### Art. VII.

*Ueber das Verhältniss der Medicin zur Chirurgie; und die Duplicität im ärtzlichen Stande, &c.* Von Dr. Ph. Fr. von Walther.—*Carlsruhe und Freiburg*, 1841. 8vo, pp. 48.

*On the Relation of Medicine to Surgery; and on the Division of Medical Practitioners into two Grades, &c.* By Dr. Ph. Fr. von Walther, Physician in ordinary to the King of Bavaria, Professor in the University of Munich, &c.—*Carlsruhe and Freiburg*, 1841.

It is certainly encouraging to medical reformers, and especially to those who advocate the reunion of medicine and surgery, to find two physicians to royalty in their ranks, namely, Sir James Clark, Bart., and Dr. von Walther, (or rather the Right Honorable Dr. von Walther, for that gentleman is a privy counsellor), physician in ordinary to the King of Bavaria.

We propose noticing the views of Dr. von Walther at length; but to afford the means for a better understanding of them, we will state the circumstances which have led to their publication. During the end of the last century and the beginning of the present, medical reform was much agitated in Germany, and questions raised precisely similar to those now agitated in Great Britain. The propriety of reuniting medicine and surgery in teaching and practice, and of having but one degree of general education, attracted the greatest attention. In 1797 and 1798 the Electoral Academy of Useful Sciences at Erfurt published the following prize questions:

"Is it necessary and possible again to reunite medicine and surgery in study and in practice?

"What were the causes of their separation, and by what means can they be reunited?"

The arguments of thirteen candidates for the prize were in favour of the reunion of medicine and surgery, and were similar to those brought forward at the present time; while one only took up the opposite side of the question. The essay of this individual was published with sketches of the other essays sent in.* It is to be observed, however, that Dr. Jugler, the prize essayist, does not really grapple with the questions, but gives a sort of *tertium quid* of his own, so contradictory and inconsistent in its details, that it is matter for surprise how the palm could have been justly adjudged to him. Various writers followed on both sides, amongst whom Professor Reil was the most remarkable. Reil dedicated his work to the celebrated Hufeland, chief of the Medico-Chirurgical College at Berlin, and advocated with all possible enthusiasm the education of a class of illiterate practitioners for the country folk which were to be *Routiniers*. The *Routinier*, like the scientific physician, was to be naturally clever; but like the boy-mathematician, or the working land-surveyor, only able to act by rules, and not to think about them. He was to know the varieties of disease, but nothing of their causes or the *modus operandi* of his remedies. Petty surgery and practical midwifery he was to have at his fingers' ends; he was to be a good herbalist, and know cheap remedies; he was to learn something of toxicology; to know more than a smattering of materia medica, chemistry, and pharmacy, and to be well versed in the natural philosophy of man, both in health and disease. He was, of course, to have, preliminary to all this, an adequate elementary education; and, as the crowning qualification, he was to be thoroughly vulgar, and exactly resemble the common people in his dress, conversation, mode of thinking, &c., that they might have the greater respect for him.† Reil was serious, as is shown by the host of opponents he raised for

* Gekrönte Preisschrift über die von der Churfürstlichen Akademie nützlicher Wissenschaft zu Erfurt aufgegebene Frage: "Ist es nothwendig, und ist es möglich, beide Theile der Heilkunst, die Medicin und die Chirurgie, sowohl in ihrer Erlernung als Ausübung, wieder zu vereinigen? Welches waren die Ursachen ihrer Trennung, und welches sind die Mittel ihrer Wiedervereinigung?" Von J. H. Jugler, M.D. &c.—Erfurt, 1799. 8vo.

† Die Pépinière zum Unterrichte ärzlicher Routiniers, als Bedürfniss des Staats; nach seiner Lage, wie sie ist. Vom Prof. Reil.—Halle, 1804. 8vo.

several years after the date of his publication. C. W. Hufeland was one of these, and had no difficulty, as may be readily supposed, in exhibiting the absurdity of Reil's plan ; but, singularly enough, he himself a few years after, proposed that the clergy should practise medicine as *routiniers*, a folly little more absurd than the recommendation not long since advocated by a weekly contemporary, that druggists should be allowed so to practise, but a folly actually carried into effect by the Swedish Diet in 1809. In Germany, secondary schools for the education of an inferior grade of practitioners (Landärtze, country doctors,) were established at Brunswick, at Berlin, in Bavaria, and in Austria. Our author raised his voice against this system in 1807, and now after an experience of the results for thirty-four years, and with ample opportunities for observing, he comes forward and expressly declares that these inferiorly-educated general practitioners have done incalculable mischief in Bavaria; and adds, that loud complaints to the same effect are made in Prussia. It is, then, to remedy these evils and to secure for the public a supply of skilful and learned general practitioners that Dr. von Walther has in view by the publication of his pamphlet.

The leading ideas in this absurd or, we may almost say, wicked system are, that a surgeon is necessarily something distinct from a physician and of an inferior grade; that the ill-educated surgeon is quite good enough for the provincial clodhoppers; and that men of superior attainments or graduates (promovirte ärtze) could not be got to live amongst them or attend them. Dr. von Walther traces the origin of these ideas historically, and as the question is one of considerable importance in medical reform, we will trace them with him.

Dr. von Walther justly observes that medical practitioners of one kind or other are to be found in all communities. They are indigenous in those which are savage or only half-civilized, and their knowledge is indigenous too. Such was the profession in Germany, France, and Great Britain not many centuries ago. In Germany the first trace of medical practitioners, as a distinct class, appears in the thirteenth century, when the bathmen, or attendants at baths, and the barbers practised medicine, and constituted two distinct guilds or corporate bodies. It does not appear which was the superior grade, corresponding to the pure physician, we suppose the bathmen; but it is certain that they quarrelled much; were guilty of breaches of etiquette, that is, of poaching on each other's domain; formed associations; and at last in the fourteenth century, were united into one guild, becoming in fact one-faculty men; and, swallowing up the autochthonic doctors, gave their name to the united profession. To this day the country people in remote districts in Germany call their medical practitioners "Bäder" and "Feldscheerer," bathmen, and army-barbers.

These were unlearned physicians or general practitioners; men who were masters of their craft, and taught it to their apprentices; but a formidable body of learned physicians began to appear at the time the two primary constituents united into one guild, and when the knowledge of Greek literature spread from Italy through the monasteries of France and Germany. At first it was considered disgraceful to study medicine, as many people think it to be now ; but the monks found it to be profitable; so the study and practice of medicine and surgery extended amongst them in spite of the denunciations of the two popes, Alexander III. and Honorius III., until the fourth Lateran council, by special edict, positively forbid the clergy to practise surgery, and declared that every priest should be degraded who shed blood. The bathmen and barber-surgeons with their indigenous lore remained, of course, quite distinct from the monk physicians and their exotic literature; for being unacquainted with the classics, they could learn nothing of Greek or Latin medical science, and so became the inferior, because the illiterate class of practitioners; while the ecclesiastical physicians confined themselves to the neighbourhood of monasteries, the courts of princes, and to large cities, and were the doctors of the higher orders of society.

The edict against the shedding of blood was more effectual than the papal bulls; but if the *patres* could not practise surgery, the *fratres* or lay-brethren might; and so another class of practitioners sprung up, the *monk-surgeons*, of which Frère Côsme and Jacque Beaulieu, in France, and Pravetz, in Germany, were the most distinguished. The universities of Italy and Spain (to meet the new demand for surgeons created by the edict), also sent out educated surgeons as well as physicians, under the title of *Chirurgi Physici.* These took an oath not to treat inward complaints; they were generally ambulatory, and never formed themselves into guilds or colleges, but associated rather with the learned physicians, the clerical and lay university-graduates, than with the unlearned practitioner, the bathmen and barbers, who knew little of operative surgery. The masters in surgery were not numerous, and as the pure physicians now thought it undignified and degrading to practise surgery, a great deal of minor surgical practice fell into the hands of the bathmen and barbers. These retaining their guild, assumed the title of chirurgeons, to place themselves on an equality with the masters in surgery, the pure surgeons of the age. The latter were also termed "chirurgeons of the long robe," and the former "chirurgeons of the short robe," but these last still continued to practise as general practitioners, like the surgeon-apothecaries of our own times.

The modern divisions in the profession were now established, but with serious detriment to surgical science, for Vogel von der Vogelweide could find no one near the Rhine or in the neighbourhood of Worms to operate for a simple harelip; so that he was compelled to travel into Thuringia to consult a famous "cutty-sark" chirurgeon, who gave him dreadful pain, allowed him to bleed for upwards of twenty-four hours, and sent him home, more disfigured than before. Nor was this all; for the two grades of practitioners just mentioned soon came in collision, and the long-robes and short-robes, quarrelling with each other, like their predecessors, the bathmen and barbers, formed associations. But at the same time they also came into collision with the physicians, who now became their oppressive enemies, and drove them into union as one faculty. The age of guilds being passed, colleges were founded in cities where the chirurgeons of the long-robe were sufficiently numerous, as in London and Paris, for example, and educational institutions were attached to them. But these institutions derived an unlearned character from the great numerical majority of illiterate short-robes in the profession. The students took no heed to the humanities; and science, the learned languages, and the medical classics were despised. Those who have been admitted members know well that in practice this is the character of the London College of Surgeons to this day; and we are firmly of opinion that nothing has tended to degrade the surgeon in the eyes of the public so much as this want of a good preliminary education in the members of that college.

The chirurgeons of the long-robe, however, became more influential and more scientific wherever these colleges were founded; and the science and art of surgery were advanced in proportion. But in Germany the elevating element, the learned chirurgeon, was not sufficiently powerful to establish colleges, so the old barber-guilds remained, and surgical science was temporarily retarded. Little evil resulted, however, from the absence of this reintegration of the profession, for in a few years the great truth that surgery and medicine are one, arose out of the confusion; and Germany, sooner than any other country, exhibited accomplished physicians, who were also accomplished surgeons, while the more enterprising and talented of the barber-surgeons came little behind these in medical skill. Langenbeck of Göttingen, we understand, was one of the latter class, and Richter, Siebold, and Loder of the former, as also Van Swieten. Still the old divisions remained; the "feldscheerer" or barber-surgeons were uninfluenced in the provinces, and the surgical physician only partly superseded the "pures" in the towns and cities. But the establishment of the new class was a great step in the right direction, and, better still, it was the practical demonstration of a great principle. As such, it had an immediate effect, and thinking men soon saw that if such a class was valuable in towns, it would be doubly valuable in the provinces where the general practitioner was so lamentably ignorant. The outcry for an improved system of medical education to which we alluded at the commencement of our remarks, quickly followed (an outcry reechoed at the time in England), and the ideas then developed terminated in the institutions for educating that inferior class of surgical physicians for the country which Dr. von Walther declares has inflicted incalculable mischief in Bavaria. It should be here observed that these new institutions were the result of a compromise between the old learned and unlearned institutions; and that, as on previous occasions, the new received the hue of the unlearned majority.

Dr. von Walther infers from these historical facts that the profession was bipartite in Germany from extraneous circumstances, but particularly from the inequality of education in the general practitioners of the day, and the consequent division of the profession into learned and unlearned. That there was no such division in practice in ancient times is evident from the Greek and Latin classics. Hippocrates, Galen, Oribasius, Ætius, Alex. Trallianus, Paulus Ægineta, Ebn Sina, and Abulcasem, all made no distinction between medicine and surgery. It is certain that the operations of surgery, including obstetric surgery, were taught in the medical school at Alexandria; but they were taught as specialities, just as the use of the stethoscope, ophthalmic pathology, or any other special branch of medicine is taught in modern medical schools. In Germany (as we have already stated), the propriety of uniting medicine and surgery has been long practically demonstrated, and not less so in France. But it is a remarkable circumstance that the old divisions of the profession produced the same results in that country as were observed in Germany, the Officiers de Santé being an inferiorly educated grade, corresponding to the landärzte. The College of Surgeons of London is the only remains in England of the barber-guilds, and in conjunction with the College of Physicians, has contributed very materially towards maintaining the old professional grades in this country. But Dr. von Walther observes that nothing but the wildest Anglomania could recommend the adoption of such singular anomalies as are seen in England, and that to establish a college of surgeons in Germany would be actually to retrograde. We agree with Dr. von Walther. The medical profession has a higher destiny than to discover minute nervous fibrils, rectify distortions, or mutilate human beings. As regards the education of physicians and surgeons, Dr. von Walther observes that the three following propositions are generally acknowledged:

1. That an accomplished physician ought to have studied surgical operations, and, if corporeally or mentally disqualified for performing them, or if without the requisite manual dexterity, ought to have a competent knowledge of surgical diseases and of the operations by which they may be cured.

2. That a good operating surgeon must be well educated in every branch of medicine.

3. That it is impossible to have a class of persons, to whom the physician shall indicate what operations are to be performed, and who will perform them as a mere mechanic under his direction.

It will have been observed that the idea of inferiority in amount of education is attached to the word surgeon, and the cause of this will also have been evident. Now, however, the scientific surgeon ranks with the accomplished physician, and the advocates for a divided profession shift their ground, and say that an inferiorly educated practitioner is necessary for the people generally; but why this practitioner should be called a surgeon does not appear. Having demonstrated that the education of physicians and surgeons should be alike, and their academic *status* equal, Dr. von Walther proceeds to discuss this question, whether it be necessary to have practitioners for the poorer classes of inferior attainments and education. He justly remarks that the notions respecting the simplicity of the diseases to which the poor are liable—that they are more surgical than medical, and consequently that a learned physician is not necessary for them,—arise from an undercurrent of money-making aristocratic pride, and are simply the fancies of a few medical hierarchs. We believe this foolish pride has materially assisted in keeping up the evil of inferior grades, and for proof we need only refer to the conduct of the two London Colleges which are (or but the other day were) *de facto*, little more than two clubs made up of a few of these same medical hierarchs. Midwifery is an ungentlemanly branch of practice, consequently those members of the College of Surgeons who practise obstetric surgery are too degraded to be eligible for a seat in the council; but the physician declares *all* surgery to be degrading. Provincial people are vulgar, therefore the college of physicians have repeatedly declared, and still declare officially by their unrepealed regulations, that a practitioner of inferior grade and of attainments inferior to their own, is quite good enough for the vast population existing outside that charmed circle in which they themselves reside, and the radius of which is precisely seven miles. That old corporations, the lifeless *debris* of the middle ages, should cling to follies like these is not wonderful, for the attachment which all bodies of men show to their prejudices and selfish interests is always most infatuated; but, that individuals should dare to come forward and endeavour to advocate and promulgate such wretched doctrines would be incredible, if melancholy experience did not assure us that there is nothing be it ever so foolish and wicked, which has not its advocates.

Disease and death attack all men alike, whether they be rich or poor. Standing between man and his last enemy, the physician in this respect is the impersonation of that Divine Providence which is good to ALL. In the exercise then of his godlike duty he must know no difference of rank or station, nor must he think anything mean or anything degrading which enables him to fulfil satisfactorily his high mission. The pettiest and most revolting services he can render to humanity are ennobled when rendered with this object in view. The assertion that an inferior practitioner can call in the aid of a superior in cases of emergency is not valid; for in the first place, it often requires consummate skill to distinguish these cases of emergency, and, secondly, in actual practice and at the bed-side, the physician is self-dependent; he cannot await instructions from a superior officer; there he must act on his own responsibility and act too, without delay. All other subordinate professional men who receive orders from superior officers, whether in the military or civil service of the state, in the church or in the law, are educated; but the man upon whose unaided decisions the lives of hundreds may depend, need not (it is argued) have a scientific education; in other words his mind need not be cultivated, his faculties trained, his judgment enlightened. And, also, because the health and ease of man is in immediate relation with all nature, and the science of medicine thereby complicated as no other science is; it is better, say the advocates of an inferior grade, that the general practitioner, to be practically useful, should know nothing of science whatever, or at least as little as possible. By parity of reasoning a Priessnitz is a more useful physician than a Prout.

Vain pride and stupidity are so often allied in the same individual, that to demand a good preliminary education in all candidates for admission to be medical students would be useful, if the only object gained by it were the exclusion of those dunces from the profession, who are now the arrantest of quacks, and at the same time the most illiberal and most exclusive of practitioners. The individual without the mind to comprehend the physical and natural sciences ought never to be intrusted with the application of those sciences to medicine. An individual so constituted should not be permitted even to enter upon the professional curriculum of study, and so a loss (to him) of time and capital would be prevented. It is upon the mental qualifications and training that Dr. von Walther very correctly lays the greatest stress. If the physician have a scientific and classical education, while that of the surgeon is neglected, the former will be the better practitioner, even with less experience and a shorter period of professional study. The objections that well-educated practitioners will be too proud to attend the poor, or not sufficiently numerous, are easily set aside by Dr. von Walther, so far as Germany is concerned. The sons of mechanics, day-labourers, and small farmers, (as more than two thirds of the medical students in the Bavarian Universities are,) are not very likely to be overburdened with pride, especially when five sixths of them consider their profession as a mere trade by which they are to be fed. As for the fear that the supply of learned physicians would fail, it appears the graduates are so numerous that every large village might have a doctor, but for the illiterate country physicians or landärtze.

Upon taking a general view of the history and development of the profession, it appears that *inequality in education and privileges* has been the principal source of the various evils we have noticed. The tendency has continually been to the union of medicine and surgery in practice, and to equalization of rank ; the inferior class pressing upwards to get on a level with the superior, and the two classes in continual broils until that level was attained. Each time that this event took place was marked by a step forwards of the united profession in scientific knowledge and in social position ; but the united profession has invariably been temporarily depressed from the position attained by the highest grade, and its literary character has received a tincture from the lower. In proof of these statements we find the bath-men and barbers quarrelling until they form one guild ; then the short-robes or barber-surgeons, and long-robes or literate surgeons are in continual broils until a union is compelled by the attacks of their common foe, the pure physician ; and lastly, in Germany the physician and surgeon unite, and the rapid advancement of the profession is only arrested by the unwise step taken about thirty-five years ago, to provide that inferior grade of surgical physician to which the more enlightened practitioner is now opposed.

The progress of the profession in England has been similar. It is true there is and was a triple division into grades, but the two lower, the surgeon and the apothecary (or druggist) quickly coalesced, yet not until there had been warm disputes, and frequent appeals to parliament, just as the general practitioner now complains of the druggist. Still the surgeon-apothecary was an uneducated man, and the same demand for an improved system of medical education was made as we have noticed in Germany. This reform agitation was headed by Dr. Harrison and others, but their scheme of education embracing the unnatural tripartite division in the profession, necessarily fell to the ground. Nevertheless the change which had taken place on the continent went on also in England in spite of all obstacles. The pure surgeon soon equalled the physician in his preliminary education, and became, as he is now, a physician in practice, corresponding in every respect except the name, to the continental graduates. The apothecaries' act of 1815, carried into effect the principle of having an inferior grade of practitioners, already acted on in France and Germany nine or ten years previously ; and the surgeon-apothecaries were now metamorphosed into surgical physicians of an inferior class. But the tendency upwards which raised this class from shopkeepers to professional men, continued still to operate.

The apothecary examiners gradually extended their curriculum of study until, in conjunction with that of the college of surgeons, it equalled in extent the *medical* curriculum of any university whatever, and exceeded that of several. On the other hand a knowledge of the greatly augmented medical sciences was required from university graduates, rather than an acquaintance with classics and mathematics, until by the disuse of the Latin language in the examinations and the formalities of graduation, the latter process became assimilated to the examinations at "the Hall and College."

We assert, then, that in England the two grades of the profession are now nearly on a level,—are at the point of fusion, and that the struggle for equality of title, rights, and privileges is coming to a close in the establishment of one faculty. Convinced that we are right we feel anxious to point out the evils which history warns us to avoid. In the first place care must be taken to prevent the less literary and more numerous grade giving its own character to the collegiate or educational institutions of the united faculty. The course of preliminary education must be extended and most strictly insisted on. In the second place, no inferior grade of practitioners must be tolerated. If the druggist be allowed to practise petty surgery, and treat "simple" cases, as thoughtless people recommend, only a few years will elapse before he become a general practitioner; in a few years more it will be found necessary to educate him to fill an inferior grade in the profession, and in another generation he will be a surgical physician, and nearly on a level with the superior grade of practitioners. Thus the profession will pass through another cycle of strife, confusion, and interruption to the quiet progress of science, and at the termination of it, we shall only be in the position to accomplish that which may more easily be done now.

Many of those who oppose the movement towards one faculty, oppose it on the supposition that one faculty will lower the dignity of the profession, and degrade the individual members in society. We think the contrary will take place if the parallel movement towards becoming a body in the body politic be encouraged and maintained. To the corporate opponents of the one-faculty scheme, we would say that the fusion of grades now impending is really inevitable; no law can prevent it taking place, nor can any effort, however well directed and energetic; and we would seriously advise them to consult their own interest and honour, by gallantly turning round and heading the movement. Our arguments will, of course, be unheeded. "They who have power are never in time with their concessions," is the profound remark of an eminent political writer, (Professor Smyth,) and too truly applicable, we suspect, to those who have power in the medical corporations. But while we call upon medical reformers to persevere in their great undertaking, we would press on their attention the advice given to reformers by the same excellent writer, namely "to hasten on to practice and to the real wants and wishes of their fellow-citizens, *as they see them plainly existing before them*, not expecting too much from themselves or others, and above all things, losing no time."

The demand of the greater portion of the population in England is for surgical physicians who dispense their own medicines, although in many large towns the latter qualification is by no means insisted on. Now, to prevent any misconception of our meaning, in using the term "one-faculty," we would observe that we wish to express by that term an incorporation of medical practitioners, having the same or similar qualification; such a qualification as shall render every individual member of the faculty a fit and proper person to practise as a surgical physician, or in other words, as a *general* practitioner. Yet while we would have him so educated that, like the general practitioner, he might practise every branch of medicine, we would by no means interfere with the established division of labour, demanded also by the public. Consulting practitioners there will always be, as well as operating practitioners; but we would have all to commence with the same general education, as the best basis on which to build up any special branch of practice. If colleges are to be multiplied according to the divisions of professional labour, and made independent of each other, let there be as many colleges as lectureships; and let the aurists, the oculists, the dentists, chiropodists, cuppers, lithotomists, rectum-doctors, spine-tinkers, surgeons, obstetricians, physicians, &c. each have their own petty guild. There is no more reason why there should be an independent college of surgeons than an independent college of accoucheurs. Surgical diseases, properly so called, are scarcely more important and various than diseases of the eye, or of the thoracic viscera. Indeed, it is not improbable, that ere long auscultators will become as distinct a class of practitioners in large cities, as the "pure" surgeons are now; under such circumstances, why object to a college of stethoscopists?

Surely it is time that these impolitic divisions in medicine should cease. Let there be one qualification and one faculty, and within that as many subdivisions as the necessities of the public and the progress of medical science require. That these subdivisions would not be few is obvious from the example of Germany, where the qualification of graduates is uniform, but the divisions of labour much more numerous than in England. That the social rank of individuals would not be altered is certain, because even now it does not depend upon the title of the individual practitioner, but rather on extraneous circumstances, as his wealth, talents, family connexions, and the mode and sphere of his practice. Rank in society and division of labour are both clearly independent of academic qualifications. The division of labour, in teaching, depends upon the progress of science; in practice, on the progress of society in wealth and numbers. A general practitioner could live well amongst a poor and thinly-scattered population when a physician would starve; and a physician might combine operative medicine with general medicine where, as a pure physician, he would have little else to do, than to go about to cards and tea. On the other hand, a physician-accoucheur established among a suitable population, might do nothing else but attend to the ladies, and do well too. By comprising all practitioners in one faculty the equilibrium between the wants and *means* of a population, and the talents and peculiar bias of practitioners, would be established without those conflicts and heart-burnings which do and will continually occur under the present professional divisions. We say nothing of the strength to be derived from such a union, nor of the great advancement medicine would obtain from a concentration of the profession.

